# Uncovering the Effects of Different Formulae of Milk Powders on the Fecal Microorganisms and Metabolites of Bengal Tiger (*Panthera tigris* spp. *tigris*) Cubs

**DOI:** 10.3390/ani15071053

**Published:** 2025-04-04

**Authors:** Xuanzhen He, Tingting Xiao, Jing Fang, Peng Zhang, Shenghui Luo, Sufang Han, Yuansheng Wu, Lizhen Li, Zhihao Cao, Yuhan Ji, Guixin Dong, Baichuan Deng

**Affiliations:** 1 Guangdong Provincial Key Laboratory of Animal Nutrition Control, College of Animal Science, South China Agricultural University, Guangzhou 510642, China; 2 Chimelong Group Co., Guangzhou 511430, China

**Keywords:** Bengal tiger cubs, milk powder, intestinal flora, fecal metabolites

## Abstract

In order to optimize diets for Bengal tiger cubs and improve their health condition and survival rates, we conducted microbiota and metabolomics analyses on fecal samples from Bengal tiger cubs fed goat and dog milk replacer formulae. The results showed that there were significant differences in fecal microorganisms and metabolites between the two groups. At the phylum level, the major components of the microbial composition in the feces of cubs were Firmicutes, Actinobacteriota, Proteobacteria, Bacteroidota and Fusobacteriota. In addition, the abundance of gut microbiota varied significantly between the two groups of tiger cubs. The fecal microbiota of the tiger cubs fed dog milk replacer powder exhibited an increase in probiotic bacteria (*Anaerostipes* and *Clostridium_scindens*) (*p* < 0.05), and the microbial community tended to be more balanced. Metabolomics data further elucidated that feeding different milk formulae significantly affected the fecal metabolites and metabolic pathways in the Bengal tiger cubs. In the dog milk replacer powder group, 76 metabolites were up-regulated (*p* < 0.05), and 278 metabolites were down-regulated (*p* < 0.05), particularly affecting the metabolism of vitamin D3, vitamin B5, isoleucine, valine, phenylalanine and oleic acid. At the same time, 19 metabolic pathways were affected (*p* < 0.05), including the amino acid metabolism, lipid metabolism and nucleotide metabolism pathways. In conclusion, this study confirms that milk formula composition affects the gut microbiota and metabolism of Bengal tiger cubs. These findings may provide new insights into how different milk powder formulae and dietary strategies influence the regulation of gut microbiota and overall health in Bengal tiger cubs.

## Simple Summary

Captive Bengal tiger cubs in zoos are frequently abandoned by their mothers, posing a significant threat to the development and sustainable preservation of the Bengal tiger population. To optimize diet and improve the health and survival of Bengal tiger cubs, we performed microbiome and metabolomics analyses on fecal samples from Bengal tiger cubs fed goat and dog milk replacer formulae. This study confirmed that milk formula composition influences the gut microbiota and metabolism of Bengal tiger cubs. These findings may provide new insights into how different milk formulae and dietary strategies affect the regulation of the gut microbiota and overall health of Bengal tiger cubs.

## 1. Introduction

The tiger (*Panthera tigris*), the world’s largest extant cat and terrestrial predator [1,2], is listed as endangered on the International Union for Conservation of Nature Red List due to population decline, habitat shrinkage and fragmentation, food scarcity and illegal poaching [3]. Among the six extant tiger subspecies, the Bengal tiger (*Panthera tigris tigris*) has the largest population but remains one of the world’s most endangered species [4].

Currently, the majority of the Bengal tiger population resides in India, accounting for about 60% of the global wild Bengal tiger population [5]. By the end of the last century, Bengal tigers were also found in the areas bordering Tibet (China) and Nepal. There is almost no stable wild Bengal tiger population in China [6]. As a result, the conservation of Bengal tigers in China relies heavily on captive breeding and transnational cooperation. Unfortunately, Bengal tiger cubs in captivity are often abandoned by their mothers in zoos, posing a significant threat to the development and sustainability of the Bengal tiger population. Captive breeding may become a key intervention to improve the cubs’ survival. Formula feeding is an effective alternative to breast milk, delivering comparable nutrition to support the immune system and maintain basic growth. At Chimelong Safari Park (Guangzhou, China), hand-feeding with formula is used to improve the survival rates of abandoned cubs.

Various microorganisms colonize the gut and form a symbiotic relationship with the intestinal mucosa [7]. This microbiota plays a crucial role in gut function, metabolism and immunomodulation [8,9]. Studies have shown that the gut microbiota established early in life influences infant health and development [10,11]. Thus, for newborns, the balance of the gut microbiota in the early stages of life is closely linked to the maturation of physiological functions and the development of the immune system [7,12].

Conservation research on Bengal tigers is essential for the expansion of their population and the survival of cubs, with potential benefits for other tiger subspecies and many other species. In this study, we conducted the first analysis of the effects of different milk powder formulae on the gut microbiota and fecal metabolites of captive Bengal tiger cubs using 16S rRNA gene sequencing and LC-MS-based metabolomics.

## 2. Materials and Methods

All experimental procedures were approved by the Experiment Animal Ethics Committee of South China Agricultural University with the approval number 2024 G030.

### 2.1. Animals and Diet

The experimental animals were Bengal tiger cubs who were abandoned by their mothers at different times in Chimelong Safari Park and artificially fed. All cubs were raised in the same environment and feeding conditions. Five healthy cubs fed with dog milk replacer powder and five cubs fed with goat milk replacer powder were randomly selected and artificially fed from the first day following birth when they could be confirmed to have been abandoned. They were divided into the Gm group (fed with goat milk replacer powder) and the Dm group (fed with dog milk replacer powder). The brewing ratio of the two milk powders was 1:5. Body weight (BW) was measured on the first day, 12 days and 40 days after birth.

### 2.2. Fecal and Milk Powder Sample Collection

Fresh fecal samples were collected from Bengal tiger cubs. They were immediately collected in sterile collection tubes after defecation, preserved and transported on dry ice to the laboratory of the School of Animal Science, South China Agricultural University (SCAU) within 24 h of collection. Samples were stored at −80 °C for further metabolomics and microbial community analyses. Samples were also collected from Gm and Dm were nutritional component analysis.

### 2.3. Compositional Analysis of Milk Powders

Both milk powders were commercially available products (goat milk replacer powder: Jinyoukang Lamb Milk Replacer, dog milk replacer powder: PetAg puppy milk replacer). The composition of the two milk powders was determined using the LactiCheckTM-02 RapiRead Milk Composition Analyzer (Hopkinton, MA, USA), which measures the composition of milk powder by analyzing the speed at which sound waves pass through the sample. The milk powder and water were mixed in a ratio of 1:5 (dilution ratio based on feeding), stirred and analyzed using the instrument.

### 2.4. Analysis of Amino Acid Content of Milk Powder Samples

The amino acid content of both milk powder samples was determined using acid hydrolysis liquid chromatography and ultra-high performance liquid chromatography–mass spectrometry (UPLC-MS/MS, Ultimate 3000, Thermo Fisher Scientific, Waltham, MA, USA). A total of 12 amino acids were examined; however, tryptophan, methionine and cysteine could not be determined because of their degradation during acid hydrolysis. We accurately weighed 0.1 g of the sample, placed it in a stoppered anaerobic tube, recorded the weight and filled the tube with nitrogen gas for protection. We added 10 mL of 6 mol/L hydrochloric acid to the tube and then placed it in an oven at 110 °C for 22 h. After acid hydrolysis, the sample was removed, cooled, and filtered into a 50 mL volumetric flask for volume determination. The filtrate was then taken with a disposable syringe and 1–2 mL of the filtrate was passed through a 0.45 μm aqueous filter membrane in a 2 mL centrifuge tube for spare use. Then, 200 μL of filtrate was accurately extracted and transferred into a 2 mL centrifuge tube, followed by the addition of 200 μL of methanol. The mixture was vortexed, sonicated for 10 min and centrifuged at 14,500 rpm for 10 min. Subsequently, 200 μL of the supernatant was aspirated into a new centrifuge tube and dried in an oven at 55–60 °C. After drying, the sample was reconstituted with 200 μL of methanol in water, vortexed and filtered through a 0.22 μm aqueous filtration membrane into a lined injection vial, then stored at 4 °C for further analysis. Quality control (QC) samples were prepared by extracting an equal amount of liquid from the homogenate of the samples and mixing thoroughly. Gradient dilution of the standard sample (various types of amino acids) solution was performed to obtain standard curve solutions with concentrations of 25 ng/mL, 50 ng/mL, 100 ng/mL, 250 ng/mL, 500 ng/mL, 1000 ng/mL, 2500 ng/mL, 5000 ng/mL and 10,000 ng/mL, respectively.

### 2.5. 16S rDNA Gene Amplicons

Total microbial DNA was extracted from fecal samples using the E.Z.N.A.^®^ Stool DNA kit (D4015, Omega, Inc., Norcross, GA, USA), and DNA quality was checked by agarose gel electrophoresis. The DNA was then amplified to target the V3-V4 region of the bacterial 16S rRNA gene using primers 515F (5′-GTGYCAGCMGCCGCGGGTAA-3′) and 806R (5′-GGACTACNVGGGTWTCTAAT-3′) [13] with barcoding. The total reaction volume for polymerase chain reaction (PCR) amplification was 25 μL, including 25 ng of template DNA, 12.5 μL of PCR mixture and 2.5 μL of each primer. Cycling conditions were as follows: initial denaturation was carried out at 98 °C for 30 s, followed by 32 cycles at 98 °C for 10 s, 54 °C for 30 s and 72 °C for 45 s. The final cycles were carried out at 72 °C for 10 min. PCR amplification products were detected by 2% agar gel electrophoresis. PCR products were purified by AMPure XT beads (Beckman Coulter Genomics, Danvers, MA, USA) and quantified by Qubit (Invitrogen, Waltham, MA, USA). Sequencing was performed by Meiji Biopharmaceuticals (Shanghai, China). After data splitting, splicing and filtering, feature tables and sequences were obtained through denoising with DADA 2 in QIIME 2 software (2023.2) [14,15]. Clustering high-quality sequences into Amplicon Sequence Variant (The complete ASV table is provided in Supplementary Table S1). The feature abundance was normalized based on the relative abundance of each sample using the SILVA (release 138) classifier. Statistical analyses and mapping of α-diversity, β-diversity, and LEfSe were conducted on the Majorbio platform https://www.majorbio.com/web/www/index (accessed on 13 October 2024).

### 2.6. Fecal Metabolomics Analysis

Thermo Fisher Scientific UPLC-Orbitrap-MS/MS system (Q-Exactive Focus, Thermo Fisher Scientific, USA) was employed for fecal metabolic profiling with slightly modifications as an untargeted metabolomics method [16]. Quality control (QC) samples were prepared by mixing equal amounts from all samples. Fecal samples were processed following previously described methods. Bengal tiger cub samples were thawed on ice, and about 60 mg of fecal matter was weighed and transferred to a centrifuge tube. The samples were homogenized using 600 μL of methanol–water (1:1, *v*/*v*) and two magnetic beads. The centrifuge tubes were subjected to an ice bath for 10 min and then stored at −20 °C for 30 min. After standing at −20 °C, the samples were centrifuged at 14,500 rpm for 15 min at 4 °C. A 200 μL aliquot of supernatant was collected from each sample, transferred to a new centrifuge tube, and evaporated to dryness using a nitrogen blower. After drying, the samples were reconstituted with 200 μL of methanol, vortexed, sonicated in an ice bath for 10 min, and centrifuged again at 14,500 rpm for 15 min at 4 °C. The supernatant was filtered through a 0.22 μm micropore membrane and then transferred to an injection bottle with a liner tube for analysis. Raw data were processed by Compound Discoverer 2.1 software (Thermo Fisher Scientific, USA), generating a data matrix including retention time (RT), mass spectrum (*m*/*z*) and peak intensity. Metabolic profiles with a relative standard deviation greater than 30% were excluded. Metabolites were identified using the mzCloud and mzVault libraries. Metabolites with VIP > 1 and a corrected *p* < 0.05 were identified as differential metabolites. These differential metabolites were then functionally annotated using the KEGG http://www.genome.jp/kegg/ (accessed on 19 September 2024) database and mapped to KEGG pathways. Further analyses were analyzed using MetaboAnalyst 5.0.2. https://www.metaboanalyst.ca/ (accessed on 20 September 2024) and the results were displayed graphically.

### 2.7. Statistical Analysis

The raw body weight data were collected and preliminarily processed using Excel 2022, and all data were expressed as the mean ± standard error (SD ± SEM). The experimental data (milk powder amino acid content and fecal metabolites) were preprocessed using Microsoft Excel 2022 and then analyzed by one-way ANOVA using SPSS 26 software with Fisher’s LSD multiple comparison test for statistical evaluation. LEfSe analyses were performed using the nonparametric Kruskal–Wallis test (*p* < 0.05), Wilcoxon test (*p* < 0.05) and LDA linear discriminant (LDA > 2). Graphs were plotted using GraphPad Prism 10.0 software. Data from milk powder samples were averaged based on 3 replicate determinations per milk powder sample, and data variability was expressed as standard error of the mean (SEM). Statistically significant was defined as *p* < 0.05, while *p* < 0.01 was considered highly significant. Significance was calculated by plotting KEGG-enriched scatterplots using the statistical package in R software (v4.4.1) and the OmicStudio tool https://www.omicstudio.cn/tool/11 (accessed on 20 September 2024).

## 3. Results

### 3.1. Milk Powder Ingredient

The composition of the two milk formulae is presented in Table 1, highlighting their significant differences. The dog milk replacer powder has lower levels of milk protein and lactose. Table 2 showed the composition of individual amino acids as a percentage of the total amino acid content in two milk powders. The percentages of glutamic acid, histidine, tyrosine and methionine were higher in dog’s milk powder than in the goat’s milk powder, while the other amino acids present lower percentages in the dog milk replacer powder.

### 3.2. Weight Changes in Bengal Tiger Cubs

**Figure 1:**
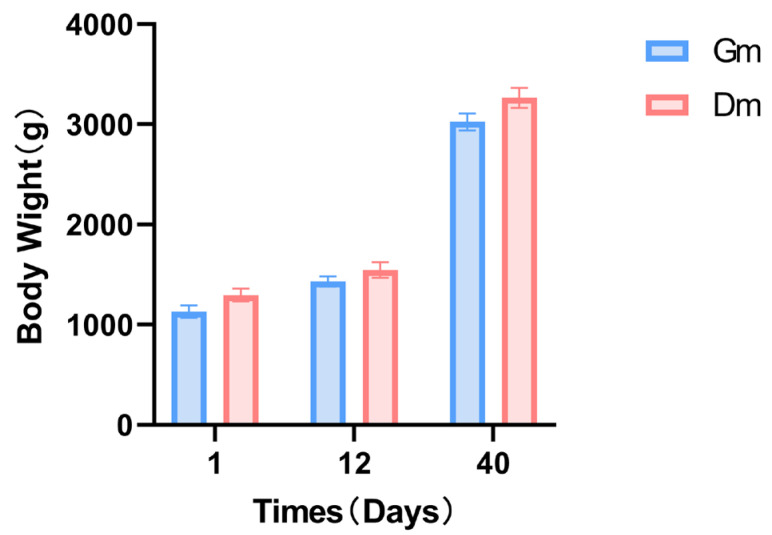
Effects of different milk powders on the body weight of lactating Bengal tiger cubs. Data are expressed as SD ± SEM. Dm: dog milk replacer powder; Gm: goat milk replacer powder. N = 5.

The weight of the pups in both groups continued to increase throughout the experiment. In contrast, there were no significant differences in weight between the two groups at any of the three time points (Figure 1).

### 3.3. Fecal Microorganisms in Bengal Tiger Cubs

**Figure 2:**
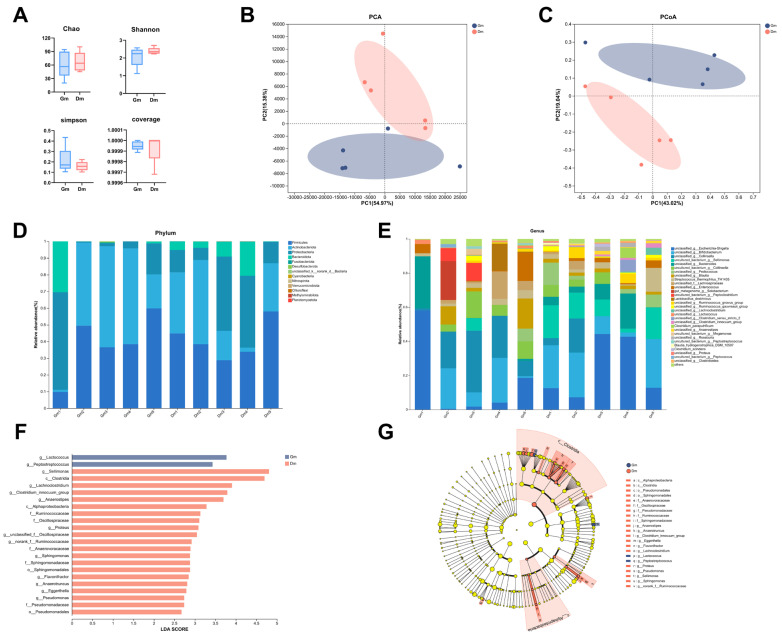
Analysis of (**A**) α-diversity and (**B**,**C**) β-diversity of fecal microorganisms. Relative abundance of fecal microflora at the level of (**D**) phylum and (**E**) genus. (**F**,**G**) LEfSe analysis between different groups. Dm: dog milk replacer powder; Gm: goat milk replacer powder. N = 5.

Analysis of the fecal microbiota of Bengal tiger cubs using 16S rDNA gene amplification revealed that a total of 763,486 high-quality read sequences were obtained for all samples, with an average of 76,348 read sequences per sample (range: 53,063–157,305). After rarefaction, the read counts between samples were comparable, minimizing potential bias in the microbiome analysis. The results showed that alpha diversity indices (Figure 2A) such as the Chao index, Shanno index, Simpson index and coverage diversity index between cubs fed different milk powders were not significantly different (*p* > 0.05). Beta diversity, measured according to the Bray–Curtis distance, showed significant separation between the Gm and Dm groups in both principal component analysis (PCA) and principal co-ordinates analysis (PCoA) plots (*p* < 0.05, Figure 2B,C), suggesting that the Gm group had a different bacterial community compared to the Dm group.

All 10 samples contained a total of 20 phyla, 37 classes, 72 orders, 126 families, and 205 genera. As shown in Figure 2D, the main phyla of the microorganisms in the feces of Bengal tiger cubs were Firmicutes, Actinobacteriota, Proteobacteria, Bacteroidota and Fusobacteriota, which accounted for >99%. The main genera, shown in Figure 2E, included *Escherichia*, *Bifidobacterium*, *Collinsella*, *Sellimonas*, *Becteroides*, *Pediococcus* and *Blautia*.

Both PCA and PCoA analyses revealed significant differences in microbial composition between the groups. Different microbial communities were identified by LEFSe analysis (Figure 2F). The results showed a significant enrichment (*p* < 0.05) of *Lactococcus* and *Peptostreptococcus* in the Gm group. In contrast, the Dm group exhibited a significant enrichment (*p* < 0.05) of *Sellimonas*, *Lachnoclostridium*, Clostridium_innocuum_group, *Anaerostipes*, *Proteus*, Oscillospiraceae_unclassified, Ruminococcaceae_norank, *Sphingomonas*, *Flavonifractor*, *Anaerotruncus*, *Eggerthella* and *Pseudomonas*.

**Figure 3:**
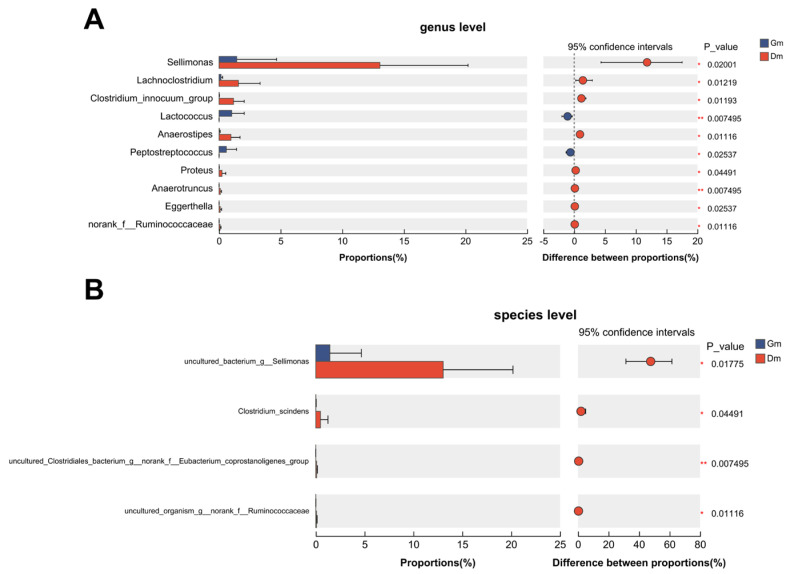
Plot of species differences analyzed between the two groups. (**A**) Differences at the genus level. (**B**) Differences at the species level. Dm: dog milk replacer powder; Gm: goat milk replacer powder. N = 5.

Ten genera showed statistically significant differences between the two groups (Figure 3). Compared with the Dm group, *Lactococcus* and *Peptostreptococcus* decreased significantly, *Sellimonas*, *Lachnoclostridium*, Clostridium_innocuum_group, *Anaerostipes*, *Proteus*, *Eggerthella* and norank_f_Ruminoccaceae increased significantly and *Anaerotruncus* increased highly significantly in the Gm group. At the species level, the Dm group showed a significant increase in uncultured *Sellimonas* species (annotated as “uncultured_bacterium_g_Sellimonas” in the SILVA database), *Clostridium_scindens* and uncultured *Ruminococcaceae* species (annotated as “uncultured_organism_g_norank_f_Ruminococcaceae” in the SILVA database), especially the uncultured *Clostridiales* bacterium (annotated as “*uncultures_Clostridiales_bacterium_g_norank_f_Eubacterium_coprostanoligenes_group*” in the SILVA database).

### 3.4. Effects of Different Milk Powders on the Fecal Metabolic Profiles of Tiger Cubs

**Figure 4:**
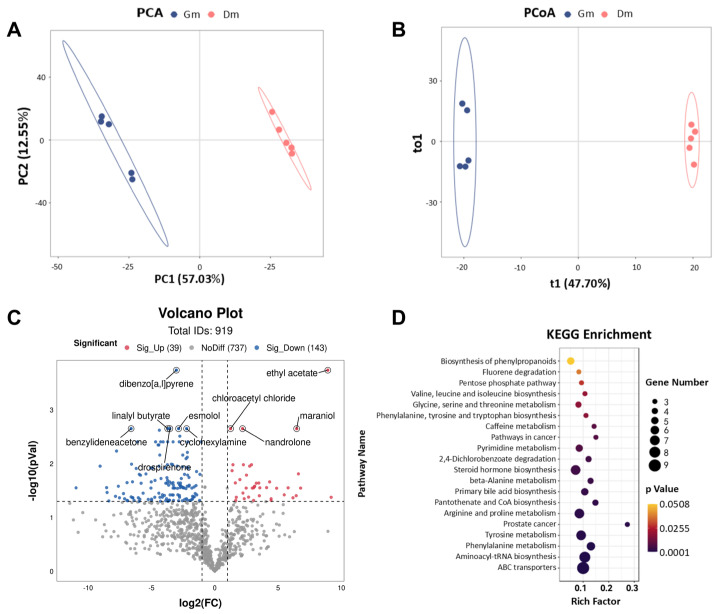
(**A**) PCA and (**B**) PCoA analysis of metabolites in the feces of Bengal tiger cubs. Effects of milk powder with different amino acid contents on the fecal metabolome of Bengal tiger cubs. (**C**) Volcano plots of the distribution of different metabolites in the two groups; blue dots represent down-regulated metabolites and red dots represent up-regulated metabolites. (**D**) Enrichment analysis of metabolic pathways in the two groups. Dm: dog milk replacer powder; Gm: goat milk replacer powder. N = 5.

In this study, fecal metabolic profiles were examined using untargeted metabolomics techniques. PCA and PCoA plots (Figure 4A,B) were employed to assess the clustering of samples. The results showed a significant separation of fecal metabolites between the two groups of tiger cubs (*p* < 0.05). Differential metabolites were identified based on the criteria of VIP > 1 and *p* < 0.05. The volcano plot (Figure 4C) showed the detection of a total of 919 metabolites in the feces of both groups of Bengal tiger cubs, with 39 fecal metabolites significantly up-regulated (*p* < 0.05) and 143 metabolites significantly down-regulated (*p* < 0.05) in the Dm group compared to the Gm group. The main metabolites included ethyl acetate, dibenzo [a, l] pyrene, cyclohexylamine, linalyl butyrate, chloroacetyl chloride, maraniol, esmolol, nandrolone, benzylideneacetone and drospirenone.

To further determine the effects of different amino acid milk powders on the metabolic pathways of tiger cubs, KEGG enrichment analysis was performed to map differential metabolites to specific metabolic pathways. Compared to Gm, Dm significantly altered 19 metabolic pathways (*p* < 0.05) (Figure 4D). These included amino acid metabolism (e.g., valine, leucine and isoleucine biosynthesis, glycine, serine and threonine metabolism, phenylalanine, tyrosine and tryptophan biosynthesis, beta-alanine metabolism, arginine and proline metabolism, tyrosine metabolism and phenylalanine metabolism), lipid metabolism (e.g., steroid hormone biosynthesis and primary bile acid biosynthesis), nucleotide metabolism (e.g., pyrimidine metabolism), translation and protein synthesis (e.g., aminoacyl-tRNA biosynthesis), xenobiotic metabolism (e.g., fluorene degradation and 2,4-dlchlorobenzoate degradation) and other metabolic pathways (e.g., pentose phosphate pathway, caffeine metabolism, pathways in cancer, pantothenate and CoA biosynthesis, prostate cancer and ABC transporters).

### 3.5. Correlation of Fecal Metabolites with Gut Microbes

**Figure 5:**
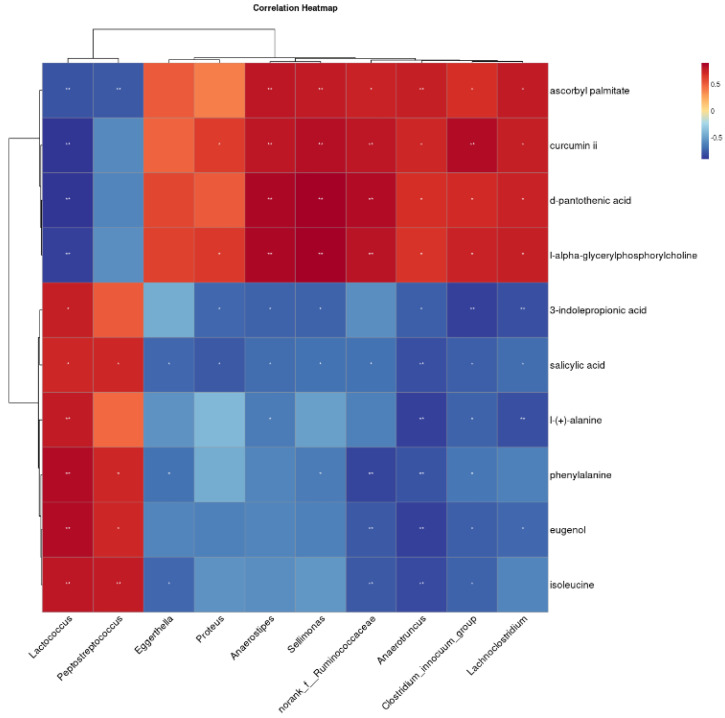
Clustering heat map of the correlation between different bacterial genera and fecal metabolites. The symbol (*) indicates a significant correlation (* *p* < 0.05 and ** *p* < 0.01).

To further explore the relationship between gut microbes and fecal metabolites in Bengal tiger cubs, Spearman’s correlation analysis was performed using differential flora and ten fecal differential metabolites known to impact organismal health. The results, displayed as heatmaps in Figure 5, revealed several significant correlations. *Peptostreptococcus* was negatively correlated with ascorbyl palmitate, and negatively correlated with salicylic acid, phenylalanine, eugenol and isoleucine. *Anaerostipes* exhibited positive correlations with ascorbyl palmitate, curcumin ii, D-pantothenic acid and L-alpha-glyceryl phosphorylcholine, while showing negative correlations with 3-indole propionic acid, salicylic acid and L-(+)-alanine. Similarly, *Lachnoclostridium* while showing negative correlations with ascorbyl palmitate, curcumin ii, D-pantothenic acid, and L-alpha-glyceryl phosphorylcholine, but negative correlations negatively correlated with 3-indole propionic acid, salicylic acid, L-(+)-alanine, phenylalanine, eugenol and isoleucine.

## 4. Discussion

With the growing concern for wildlife conservation and welfare around the world, breeding endangered species in zoos has become a vital conservation strategy. Understanding the factors that affect the survival and quality of life of animal offspring in zoo breeding can help zoos improve their husbandry management and breeding.

Infancy is a critical period for establishing the gut microbiota in animals and is also recognized as a key window for intervention [17]. The gut microbiota also provides hosts with various enzymes and additional biochemical metabolic pathways that interact with the external environment to influence host organismal function [18]. In human infants, the gut microbiota is dominated by bifidobacterial [19,20]. Diet has been shown to influence changes in the gut microbiota of infants [21], with improved diets increasing the abundance of *Bifidobacterium anisopliae* in the gut. A high abundance of *B. anisopliae* has been linked to enhanced cognitive and language development in infants [22], whereas a lower *B. anisopliae* abundance is highly associated with inflammatory bowel disease [23]. It is, therefore, important to explore the effects of different diets on the gut microbiology of cubs across various species. While numerous studies have examined the gut and fecal microbiota of adult tigers across subspecies, including northeastern tiger cubs and South China tiger cubs [24,25,26], the fecal microbiota and metabolites of Bengal tiger cubs remain largely unexplored.

The aim of this study was to analyze the effects of different amino acid milk powders on the gut microbial composition and fecal metabolites of Bengal tiger cubs. For the first time, 16S rRNA gene sequencing and LC-MS-based metabolomics were employed to evaluate the impact of different formulae on gut flora and fecal metabolites of captive Bengal tiger cubs. The results showed that different milk powders significantly affected the gut microbial community composition in Bengal tiger cubs. Moreover, significant changes in fecal metabolites and affected metabolic pathways were observed in tiger cubs in the Dm group compared to the Gm group.

The weight data indicated no significant differences between the two groups at any of the three time points (*p* > 0.05). Previous studies suggest that kittens experience rapid growth between 2 and 8 months of age [27], with research on lynx identifying the peak growth rate between 61 and 80 days [28]. Our study, however, focused solely on lactating tiger cubs, whose weight at 40 days remained relatively low. Therefore, we can infer that the observed differences in intestinal microorganisms between the two groups are likely attributable to variations in formula diets rather than differences in growth rates.

We analyzed the composition of the goat and dog milk replacer powders. The results showed that the dog milk replacer powder had a lower level of lactose and a higher level of glutamate, histidine, tyrosine and methionine compared to the goat milk replacer powder. A high lactose intake is one of the causes of lactose intolerance [29,30]. There are numerous studies showing that lactose intolerance causes diarrhea in infants, adults and other animals [31]. Milk protein allergy has been shown to induce diarrhea in infants [32]. Furthermore, research has demonstrated that artificial milk powder low in histidine may lead to strabismus, cataracts and skin problems in Bengal tigers [33]. Additionally, formulae with low histidine may lead to eye and skin problems in this species.

Intestinal inflammation may be caused by variations in intestinal flora. In the present study, the dominant fecal microbial species in Bengal tiger cubs were identified as Firmicutes, Actinobacteriota, Proteobacteria, Bacteroidota and Fusobacteriota. These findings are consistent with previous research on the fecal microflora of tiger cubs from other tiger subspecies. For instance, in South China tiger cubs, Proteobacteria and Firmicutes were found to dominate the gut microbiota [26]. Similarly, in Northeastern tiger cubs, Firmicutes, Bacteroidota. Proteobacteria, Actinobacteriota and Fusobacteriota were reported to have the highest relative abundances [24].

The results showed that there were significant differences in the abundance of genera in the intestinal microbial community of tiger cubs fed different milk powders. In the Dm group, the proportion of beneficial microorganisms, such as *Anaerostipes*, norank_f_Ruminoccaceae, *Lachnoclostridium* and *Clostridium_scindens* was significantly higher (*p* < 0.05). *Anaerostipes* is a producer of butyric acid and one of the most efficient digestors of lactic acid in the human gut [34]. Research has demonstrated that *Anaerostipes* can convert inositol to propionate in an anaerobic conditions, and the combined action of *Anaerostipes* and inositol has been associated with reduced fasting blood glucose levels in mice [35]. *Lachnoclostridium* is known to produce short-chain fatty acids and has been positively correlated with body weight in mice [36]. This suggests that the increased abundance of *Lachnoclostridium* may improve the digestive and absorptive functions of the host intestine, and enhance the transport of proteins and nutrients [37,38]. In contrast, the abundance of pathogenic bacteria such as *Peptostreptococcus* was significantly lower in the Dm group. *Peptostreptococcus* has been shown to cause pyoderma in dog cubs [39]. Another group of researchers found that tiger cubs consuming goat’s milk powder were more susceptible to skin diseases [40]. Our results showed a significantly lower abundance of *Peptostreptococcus* in the Dm group, suggesting potential for skin protection.

Metabolites derived from gut microbiota are closely related to nutritional status and metabolism functions of the host organism [41]. Using untargeted metabolomics, we analyzed the feces of 10 Bengal tiger cubs, revealing significant separation between the Gm and Dm groups in the PCA plots. The dog milk replacer powder exhibited 76 upregulated metabolites. KEGG enrichment analysis showed that dog milk replacer powder significantly altered nineteen metabolic pathways compared to goat milk replacer powder, including eight related to amino acid metabolism, two to lipid metabolism, one to nucleotide metabolism, one to translation and one to nutrient metabolism, one to metabolic metabolism and one to nucleotide metabolism, one to translation and protein synthesis, two to Xenobiotic metabolism and five to other metabolic pathways. Dog milk replacer powder primarily affects amino acid metabolic pathways in tiger cubs, with specific upregulation of, pathways related to phenylalanine, tyrosine, glycine, serine and threonine metabolism.

Phenylalanine, one of the precursors of tyrosine, directly influences brain-derived neurotrophic factors, thereby enhancing memory. Recent studies have shown that tyrosine enhances energy metabolism and delays fatigue [42]. Additionally, research suggests that selective serotonin reuptake inhibitors attenuate spontaneous scratching behavior in chronically itchy mice [43], while dietary supplementation with tryptophan reduces territorial aggression in dogs fed a low-protein diet [44]. A study on amino acid level changes in an obese population found that 15 amino acids were reduced in the serum metabolites in the metabolic syndrome cohort compared to the general obese population. The arginine and proline metabolic pathways were also significantly altered [45]. In Bengal tiger cubs, feeding with dog milk replacer formula significantly increased fecal levels of phenylalanine, tyrosine and tryptophan, affecting amino acid metabolic pathways. This effect may be attributed to specific components of the dog milk replacer powder formula. However, further research is needed to understand the relationship between different formulae and fecal metabolites fully.

Correlation analyses demonstrated a significant relationship between gut microbiota and fecal metabolites in the tiger cubs, suggesting that different formulations may influence health by modulating gut microbiota composition and its metabolites. However, further studies are required to elucidate the specific effects of these changes on the health of tiger cubs.

The key limitation of this study is that only one fecal sample was tested per individual. Single-time point sampling is a widely accepted approach in microbiome studies, especially in dietary intervention studies. Given that all cubs were on a controlled diet and were at similar developmental stages, we aimed to capture representative microbial differences between the two groups at the selected time points. Nonetheless, we acknowledge that repeated sampling could provide more insights into intra-individual variation and microbiome stability.

The exact nutritional requirements of tiger cubs between birth and 40 days of age are unknown and were not compared with cubs raised by their mothers. Further studies are needed to determine the nutritional requirements of tiger cubs during lactation and the ideal nutritional composition of milk replacers.

## 5. Conclusions

This study represents the first analysis of the effects of different formulae of milk powders on the gut flora and fecal metabolites of captive Bengal tiger cubs using 16S rRNA gene sequencing and LC-MS-based metabolomics.

The two different formulae did not affect the growth weight of Bengal tiger cubs. Feeding with dog milk replacer formula significantly increased the number of probiotics and promoted a more balanced microbial community. KEGG enrichment analysis further clarified that the different formulae altered fecal metabolites and metabolic pathways. In summary, these findings highlight the impact of feeding with different formulae on the health of Bengal tiger cubs, provide new insights into gut microbiota regulation and provide perspectives on the role of dietary strategies in the health and physiology of Bengal tigers.

## Figures and Tables

**Table 1:** Nutritional composition of milk powder.

Index	Goat Milk Replacer Powder	Dog Milk Replacer Powder
Milk fat (g/100 mL)	4.73	6.01
Milk protein (g/100 mL)	4.80	3.61
Lactose (g/100 mL)	6.94	5.19

**Table 2:** Percentage composition of amino acids to total amino acids in different milk powders.

Index	Goat Milk Replacer Powder	Dog Milk Replacer Powder
Lysine	14.11%	12.04%
Arginine	5.58%	5.24%
Glycine	2.93%	2.32%
Serin	6.91%	5.82%
Threonine	11.65%	8.55%
Glutamic acid	43.73%	45.68%
Proline	0.03%	0.03%
Valine	4.28%	3.88%
Histidine	3.09%	3.67%
Tyrosine	3.59%	5.71%
Methionine	4.09%	7.05%
Tryptophan	0.0005%	0.0005%

## Data Availability

The data presented in this study are available in the manuscript.

## Supplementary Materials

The following supporting information can be downloaded at: https://www.mdpi.com/article/10.3390/ani15071053/s1, Table S1: ASV abundance and taxonomic classification in fecal samples from Bengal tiger cubs.
